# Preoperative evaluation of endoscopic submucosal dissection for early gastric cancer

**DOI:** 10.1097/MD.0000000000030582

**Published:** 2022-09-16

**Authors:** Yu-Lan Gao, Yue-han Zhang, Meng Cao

**Affiliations:** a Department of Gastroenterology, Hebei General Hospital, Shijiazhuang, Hebei, China.

**Keywords:** Early gastric cancer, endoscopic submucosal dissection, high grade intraepithelial neoplasia, preoperative evaluation

## Abstract

To retrospectively analyze the preoperative endoscopic evaluation of the size, nature, and depth of lesions in endoscopic submucosal dissection (ESD) for early gastric cancer and precancerous lesions, determine whether the lesions can be completely resected, and reduce the risk of additional surgery after ESD.

**Methods:**

A total of 114 patients with high-grade intraepithelial neoplasia(HGIN) and early gastric cancer treated with ESD in Hebei General Hospital from January 2016 to April 2021 were enrolled in this study. The lesions were evaluated preoperatively according to the endoscopic findings of white light, magnifying endoscopy, endoscopic features of narrow band imaging, and preoperative pathology. Lesion size, positive resection margin, lesion depth, and vascular invasion of postoperative pathology were used as criteria.

**Results:**

There were 121lesions in 114 patients. The coincidence rates of preoperative and postoperative pathology were 87.21% (75/86) for HGIN and 92.1% (35/38) for adenocarcinoma. There was no significant difference in the coincidence rate between preoperative pathological evaluation and postoperative pathology among the 3 lesions (*χ*2 = 10.614, *P* = .005). The type and malignancy of the lesion were not related to its location or size. Magnifying endoscopy combined with narrow-band imaging showed that HGIN and early gastric cancer had clear borders, irregular microvessels, and irregular surface microarchitecture on endoscopic features. Lesions > 3 cm, surface ulcers and spontaneous bleeding may be risk factors for deeper lesions.

**Conclusion:**

ESD is not only a method for the treatment of early gastric cancer and precancerous lesions, but is also an important method for definite pathological diagnosis. Accurate preoperative assessment of lesion type, lesion extent and depth of invasion is helpful to improve the complete resection rate of ESD and reduce the risk of additional surgery.

## 1. Introduction

Early gastric cancer is defined as cancer tissue confined only to the mucosa or submucosa regardless of the presence of lymph node metastasis.^[1]^ Precancerous lesions refer to pathological changes that have been confirmed to be closely related to the occurrence of gastric cancer, that is, intraepithelial neoplasia, which is divided into low-grade intraepithelial neoplasia (LGIN) and high-grade intraepithelial neoplasia (HGIN). According to the 2015 guidelines for the treatment of gastric cancer in Japan, approximately 25.0% of patients with HGIN will progress to adenocarcinoma within 1 year of onset.^[2]^ At present, with the update of endoscopy and treatment equipment and the improvement of operation technology, endoscopic treatment of early gastric cancer is possible. At the same time, people’s health awareness is gradually improved, and the detection rate of early gastric cancer is increasing year by year. Endoscopic submucosal dissection (ESD) is the standard for the treatment of early gastric cancer, which has the advantages of high complete resection rate, less trauma, rapid recovery and low cost, and can also be used as a diagnostic method.^[3,4]^ It is the preferred regimen for differentiated intramucosal carcinoma without ulceration as well as undifferentiated intramucosal carcinoma <2 cm in diameter.^[5]^ However, the inaccurate evaluation of the type, depth, and extent of the lesion may leads to the failure of ESD treatment, additional surgery, and increased doctor-patient disputes, so ESD is very important for the evaluation of the size, nature, depth, border, and difficulty of complete resection of the lesion, and this paper retrospectively analyzes the preoperative evaluation of ESD.

## 2. Materials and Methods

### 2.1. General information

A total of 114 patients with gastric mucosal HGIN and early gastric cancer who were treated in Hebei General Hospital from January 2016 to April 2021 were selected as the study subjects. There were 79 males and 35 females, aged 40 to 82 years, with mean one of (60.21 ± 6.98) years. Inclusion criteria: ① patients with mucosal lesions by gastroscopy and no lymph node metastasis by CT; ② patients with ESD indications; ③ patients who voluntarily participate in this study and have signed the informed consent form. Exclusion criteria: ① only gastroscopy, without subsequent ESD treatment; ② diagnosed as advanced gastric cancer by white light endoscopy. The endoscopy and records of all patients were retrospectively analyzed, including patient basic information, clinical symptoms, conventional white light endoscopy, narrowband imaging (NBI), magnifying endoscopy, histopathological features, surgery-related adverse events, and postoperative results during follow-up. Before endoscopy, informed consent was obtained from all patients and approved by the hospital ethics committee.

### 2.2. Method

#### 2.2.1. Main instruments.

GIF-260H, GIF-290H gastroscope, GIF-260Z magnifying gastroscope, injection needle, Dual knife and hemostatic forceps (Olympus, Japan).

#### 2.2.2. Endoscopy.

10 minutes before endoscopy, the patient orally took tetracaine mucilage, used sodium bicarbonate, dimethicone and pronase to remove the foam and mucus. First, used the common endoscopic mode for examination, carefully observed the local mucosa different from the surrounding mucosal manifestations, such as local mucosal color changes (redness or whitening), local mucosal fine granular or small nodular roughness, local mucosal elevation or depression, superficial mucosal erosion or ulcer, submucosal vascular network disappeared, mucosal folds interrupted or disappeared, mucosal tissue fragility, easy spontaneous bleeding, surrounding mucosal concentration or mucosal bridge formation and other abnormal manifestations. Second, magnifying endoscopy and NBI examination, carefully observed the surface microstructure of the lesion, including the presence or absence of boundary lines, irregular mucosal microvessels and irregular surface microstructure.

#### 2.2.3. Endoscopic classification.

Endoscopic classification of early gastric cancer was based on the 2005 Paris classification criteria. Superficial gastric cancer (type 0) is divided into elevated lesions (0-I), flat lesions (0-II), and depressed lesions (0-III). Type 0-I is subdivided into ingrained (0-Ip) and uningrained (0-Is). Type 0-II is divided into 3 subtypes: 0-II a, 0-II b, and 0-II c according to the slightly elevated, flat, and slightly depressed lesions.

### 2.3. Outcome measures

Lesion size, white light endoscopic findings, magnifying endoscopy, NBI endoscopic findings, postoperative pathological type, positive margin rate, incidence of bleeding, infection, and perforation.

### 2.4. Statistical methods

SPSS 19.0 software was used for statistical analysis. Measurement data not conforming to normal distribution were described in the form of M (Q1, Q3). Wilcoxon signed rank test was used for comparison. Categorical data were described in the form of number of cases (%). *χ*^2^ test for continuous correction or Fisher exact test were used for comparison. *P* < .05 was considered statistically significant.

## 3. Results

### 3.1. Location of each lesion

A total of 121 lesions were found in 114 patients: 13 (10.74%) in the gastric body, 15 (12.40%) in the gastric angle, 28 (23.14%) in the antrum, and 65 (53.72%) in the cardia and subcardia.

### 3.2. Preoperative assessment of the nature of the lesion and diagnosis of postoperative pathological findings

**Figure 1.**
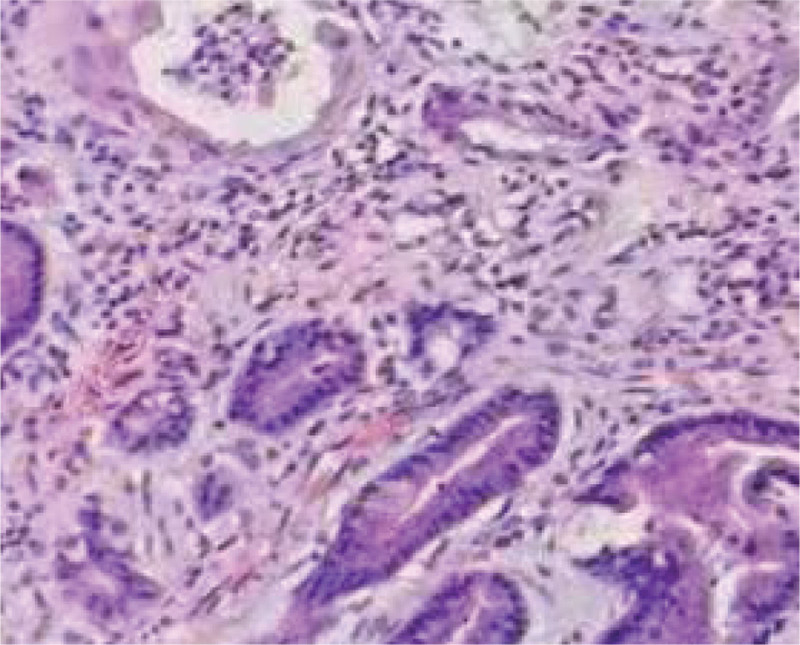
high-grade intraepithelial neoplasia (HE staining × 100).

**Figure 2.**
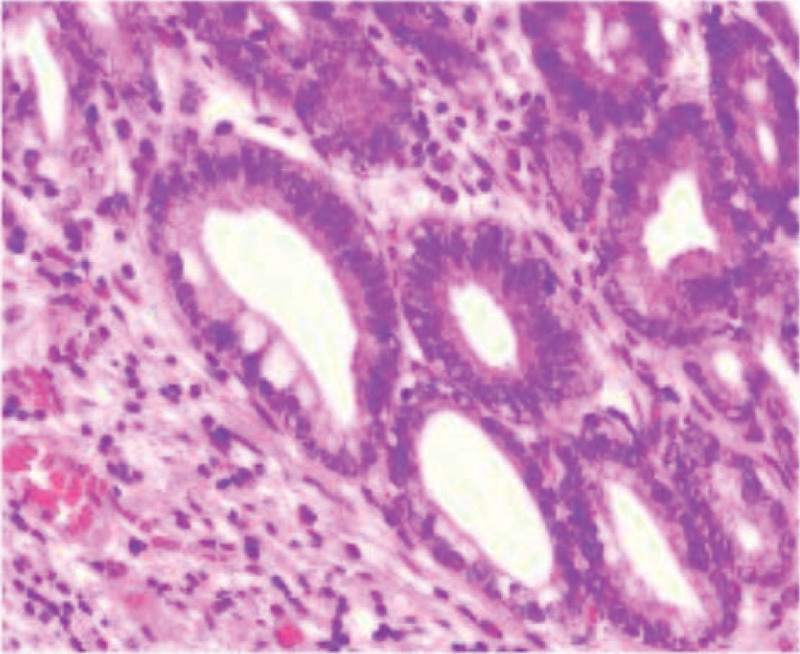
low-grade intraepithelial neoplasia (HE staining × 100).

The nature of the lesions was assessed preoperatively as 0 for LGIN, 86 (71.07%) for HGIN, and 35 (28.93%) for adenocarcinoma. Postoperative pathological diagnosis was LGIN (Fig. 1) in 8 (6.61%), HGIN (Fig. 2) in 75 (61.98%), and adenocarcinoma in 38 (31.40%). The preoperative and postoperative pathological coincidence rates were 0% (0/8) for LGIN, 87.21% (75/86) for HGIN and 92.1% (35/38) for adenocarcinoma. The postoperative pathology was milder than the preoperative pathology in 6.61% (8/121), and the postoperative pathology was heavier than the preoperative pathology in 2.48% (3/121). There was no significant difference in the agreement rate between preoperative pathological assessment and postoperative pathology among the 3 lesions (*χ*^2^ = 10.614, *P* = .005).

### 3.3. Preoperative depth assessment

Preoperatively, 117 lesions were confined to the epithelial layer, lamina propria, and mucosa by white light lesion characteristics, magnifying endoscopy, and indigo carmine staining, and the possible depth of invasion was considered to reach 4 superficial submucosa. Postoperative pathological results showed that the depth of invasion was limited to 116 sites in the epithelial layer, lamina propria, and mucosa, and the depth of invasion reached 5 sites in the submucosa. Among them, 3 cases were inconsistent, 1 case was considered to infiltrate the submucosa, but postoperative pathology was limited to the mucosal layer, 2 cases were limited to the mucosal layer by preoperative evaluation, and postoperative pathology revealed submucosal invasion of the lesion, with a depth accuracy of 97.52% by preoperative evaluation. There were 5 lesions infiltrating into the submucosa, of which 2 cases of 0-IIb lesions, both <2 cm, one of which was located in the angle of the stomach, showing large nodules in the depression, surrounding concentrated fold swelling, and mucosal bridge formation. One was located in the posterior wall of the cardia, with interruption of the surrounding mucosal elevation and central depression erosion and ulcer. In 3 cases of type 0-IIc lesions, lesions > 3 cm, surface ulcers and spontaneous bleeding may all be risk factors for deeper lesions (Table 1).

**Table 1:** Relationship between endoscopic performance and postoperative lesion depth [n (%)].

Item	Mucosal layer and mucosa (n = 116)	Submucosal layer even deeper (n = 5)
LGIN	8 (6.90)	0 (0)
HGIN	75 (64.65)	0 (0)
Adenocarcinoma	33 (28.45)	5 (100.00)
Concurrent ulcer	16 (13.79)	3 (60.00)
Spontaneous hemorrhage	17 (14.66)	3 (60.00)
Peripheral fold accumulation	3 (2.59)	2 (40.00)
nonextension sign positive	10 (8.62)	2 (40.00)
Mucosal bridge formation	0 (0)	2 (40.00)

LGIN = low-grade intraepithelial neoplasia, HGIN = high-grade intraepithelial neoplasia.

### 3.4. Endoscopic characteristics of 121 lesions and assessment of lesion types by magnifying endoscopy + NBI

According to the 2005 Paris classification criteria, there were a total of 121 lesions, mostly type 0-II lesions, 108 (89.26%) in total, type 0-IIa lesions in 21 (17.36%), type 0-IIb lesions in 42 (34.71%), and type 0-IIc lesions in 45 (37.19%), and the endoscopic findings were mainly redness, clear borders, irregular surface microarchitecture, and irregular microvessels (Tables 2 and 3).

**Table 2:** Endoscopic features of 79 early gastric cancers [n (%)].

Item	Type 0-I		Type 0-II			0-III (2)	Total (121)
	Type 0-Ip (1)	0-Is type (10)	0-IIa (21)	0-IIb (42)	0-IIc (45)		
Redness	1 (100.00)	8 (80.00)	17 (80.95)	29 (69.05)	41 (91.1)	2 (100.00)	98 (80.99)
Whitish	0 (0)	1 (10.00)	2 (9.52)	11 (26.19)	3 (6.67)	0 (0)	17 (14.05)
Tone change yellow	0 (0)	2 (20.00)	13 (61.90)	19 (45.24)	15 (33.33)	0 (0)	49 (40.50)
Clear boundary	1 (100.00)	10 (100.00)	20 (95.24)	39 (92.86)	41 (91.11)	2 (100.00)	113 (93.39)
Irregular microstructure	1 (100.00)	8 (80.00)	18 (85.71)	41 (97.61)	42 (93.33)	2 (100.00)	112 (92.56)
Microvascular irregularities	1 (100.00)	9 (90.00)	18 (85.71)	41 (97.61)	41 (91.11)	1 (50.00)	111 (91.74)
Atrophy/bowel background	1 (100.00)	4 (40.00)	12 (57.14)	38 (90.48)	32 (71.11)	0 (0)	87 (71.90)
WOS*	0 (0)	3 (30.00)	9 (42.86)	21 (50.00)	9 (20.00)	0 (0)	42 (34.71)
Concurrent ulcer	0 (0)	2 (20.00)	5 (23.81)	7 (16.67)	4 (8.89)	2 (100.00)	20 (16.53)
Spontaneous hemorrhage	0 (0)	3 (30.00)	4 (19.05)	4 (9.52)	8 (17.78)	2 (100.00)	21 (17.36)
Peripheral fold accumulation	0 (0)	0 (0)	0 (0)	1 (2.38)	2 (4.44)	0 (0)	3 (2.48)

Note: Select the most important classification for lesion classification.

* WOS = white opaque substance.

**Table 3:** Magnifying endoscopy + NBI findings compared with lesion type [n (%)].

Pathological classification	N	Magnifying Endoscope + NBI		
		Clear boundary	Irregular surface microstructure	Irregular vessel
LGIN	8	6 (75.00)	4 (50)	4 (50)
HGIN	75	72 (96.00)	73 (97.33)	71 (94.67)
Adenocarcinoma	38	35 (92.11)	35 (92.11)	36 (94.74)
X2		5.311	23.541	19.682
P		0.070	<0.001	<0.001

LGIN = low-grade intraepithelial neoplasia, HGIN = high-grade intraepithelial neoplasia.

## 4. Discussion

Clinically, common gastroscopy white light examination is still the main method for the diagnosis of gastric cancer. However, early gastric cancer and precancerous lesions are not typical under common gastroscopy white light, the boundary of the lesion is not easy to distinguish, the arbitrariness of examination is large, and even some lesions are not easy to be detected, and the positive rate of pathological examination is low. At present, with the development of endoscopic technology, there has been a breakthrough in the early diagnosis and treatment of gastric cancer.^[6]^ NBI and magnifying endoscopy rely on a combination of spectra to visualize subtle changes in the vascular and mucosal surfaces, achieving endoscopic “light staining.” By observing changes in gastric mucosal tone, smooth surface, lesion morphology, and lesion borders, targeted biopsy guided by ME-NBI can improve the detection of dysplasia.^[7]^ NBI combined with endoscopy is simple and safe and effective, and improving the accuracy of biopsy pathological examination while accurately discerning early gastric cancer and precancerous lesions has important clinical significance for early diagnosis and timely treatment of early gastric cancer and precancerous lesions.^[8]^ Indigo carmine staining and acetic acid staining can clearly show the changes in the contour, border, and surface structure of early gastric cancer and precancerous lesions, improving the diagnostic rate of early gastric cancer.^[9–11]^ Newly developed focused laser endomicroscopy allows biopsy imaging of the gastric mucosal surface during gastroscopy, providing a rapid and reliable diagnostic tool for in vivo histological studies. In the diagnosis and treatment of early gastrointestinal cancer, digestive endoscopy is not only a diagnostic technique but also a therapeutic technique, which can improve the detection rate and therapeutic effect of early gastrointestinal cancer.^[12]^

The results of this study showed that there was no significant correlation between the type of lesion and the location of the lesion, but there was a significant correlation with the maximum long diameter of the lesion and the lesion area. Magnifying endoscopy combined with NBI observation showed that high-grade intraepithelial neoplasia and early gastric cancer were characterized by clear boundaries, irregular microvessels, and irregular surface microarchitecture. This study showed that the lesions invaded the submucosa in 5 patients and underwent surgery or chemoradiotherapy, with a better prognosis, and the causes of deeper lesions may be large nodules in the depressions, swelling of the surrounding concentrated folds, embankment elevation of the surrounding mucosa, central depressed erosion, and spontaneous bleeding.

After ESD treatment of early gastric cancer and precancerous lesions, there is still a certain chance of local recurrence. Lesion size, operation time, incomplete resection, pathological diagnosis, and atypical morphology of scars after ESD are predictors of recurrence after ESD treatment in early gastric cancer and precancerous lesions.^[13]^ However, accurate preoperative assessment of lesion type, lesion extent, and depth of invasion is beneficial to improve the complete resection rate of ESD and reduce the risk of postoperative recurrence. Several studies have treated submucosal invasive early gastric cancer with ESD, which preserves the stomach in 28.8% of patients, while ESD does not affect the results of further subtotal gastrectomy surgery. Diagnostic ESD should be regarded as a treatment modality in some cases of submucosally infiltrating gastric cancer.^[14,15]^

In summary, gastric HGIN and early cancer can be treated with ESD, which has the advantages of complete resection rate, less trauma, fewer postoperative complications, high quality of life, and less cost.^[16,17]^ Accurate preoperative assessment of lesion type, extent of disease, and depth of invasion is beneficial to improve the complete resection rate of ESD and reduce the risk of additional surgical procedures. Therefore, precise preoperative assessment is necessary. This study had the following limitations: (1) few cases, especially those with submucosal invasion, which required more cases to be accumulated; (2) this study was a single-center retrospective analysis, and the results were biased. However, we can still summarize the importance of preoperative evaluation of ESD, which needs to be comprehensively judged in combination with white light endoscopy, chromoendoscopy, magnifying endoscopy, and preoperative biopsy pathology.

## Author contributions

Yu-Lan Gao and Meng Cao: 1. Substantial contributions to the conception and design of the work; 2. Drafting the work or revising it critically for important intellectual content;

Yue-han Zhang: Substantial contributions to the acquisition, analysis, or interpretation of data for the work and the analysis and manuscript preparation.

Yu-Lan Gao: 1. Final approval of the version to be published; 2. Agreement to be accountable for all aspects of the work.
